# Proactive control of proactive interference using the method of loci

**DOI:** 10.5709/acp-0156-3

**Published:** 2014-06-09

**Authors:** Willa S. Bass, Karl M. Oswald

**Affiliations:** Department of Psychology, California State University, Fresno, USA

**Keywords:** method of loci, proactive interference, mnemonics

## Abstract

Proactive interferencebuilds up with exposure to multiple lists of similar items with a resulting reduction in recall. This study examined the effectiveness of using a proactive strategy of the method of loci to reduce proactive interference in a list recall paradigm of categorically similar words. While all participants reported using some form of strategy to recall list words, this study demonstrated that young adults were able to proactively use the method of loci after 25 min of instruction to reduce proactive interference as compared with other personal spontaneous strategies. The implications of this study are that top-down proactive strategies such as the method of loci can significantly reduce proactive interference, and that the use of image and sequence or location are especially useful in this regard.

## Introduction

*Proactive interference* is the disruptive effect of prior learning on the recall of information learned more recently (Keppel & Underwood, 1962; Lustig & Hasher, 2002) and is considered a main cause of forgetting (Underwood, 1957). In the classical view, proactive interference (PI) results from response competition between target and non-target information at recall (Postman & Underwood, 1973). Underwood (1957) noted that recall from long-term memory declines across successive lists, which was attributed to increasing competition of multiple associations at recall (Wickens, Moody, & Dow, 1981). However, PI can be reduced through proactive control when participants are instructed to selectively ignore non-target information (Bjork & Bjork, 1996; Gazzaley, Cooney, Rissman, & D’Esposito, 2005). Additionally, by engaging in controlled retrieval strategies, PI is reduced in some younger adults (Ikier, Yang, & Hasher, 2008), suggesting that intentional, proactive control can reduce PI.

The current study examines whether proactive use of the *method of loci* (MOL) - a powerful mnemonic technique known to enhance recall through distinctive encoding - reduces PI relative to spontaneous encoding/retrieval strategies. To date, research on proactive control in reducing interference concerns ignoring information rather than focusing on it. The MOL is an organizational strategy that relies on encoding information by using sequential loci and mental images (see Bellezza, 1996). It provides an immediate form of memory, one that can hold any concrete information and make it available while retaining serial order. Each locus holds one piece of information, and each piece is retrieved one at a time by mentally moving from one locus to the next, thus enhancing distinctive encoding through unique cues of both image and location. In *De Oratore* (55 BC), Cicero refers to the two-pronged structure of the method of loci: place or loci to preserve the sequential order of facts, and image to preserve facts themselves (Yates, 1966). While the method of loci evolved as a strategy especially adapted to the needs of oral discourse, it is well known to improve word recall and is commonly used by memory experts in mnemonic competition. We used the method of loci as a mnemonic strategy because it enhances distinctive encoding while potentially reducing retrieval competition, both of which have been shown to reduce PI in previous research.

Massen and Vaterrodt-Plünnecke (2006) questioned whether repeated use of the MOL would cause significant PI, thereby reducing its usefulness over time. They found that the MOL retains its effectiveness and that the effect of PI was minimal. Their question focused on repeated use of MOL whereas our research investigates whether a single use of the MOL for 25 items reduces PI relative to no strategy instructions.

Classical interference theory suggests that a lack of distinctive encoding may set the stage for the development of interference. *Encoding* is the integration of information into long-term memory which occurs by converting an item into a construct that can be stored along with specific cues to facilitate recall (Wickens, Dalezman, & Eggemeir, 1976). Items are encoded in a specific way, and cues that are effective at retrieval must reflect that specificity (Tulving, 1983). When specificity is lost - such as when a cue needed to retrieve one item becomes associated with many similar items - PI may result, thereby making retrieval more difficult (Anderson & Neely, 1996; Van Dyke & McElree, 2006; Wickens et al., 1976). Jenkins (1974) recognized the importance of context cues in memory and attributed the memory decline associated with PI to superficial encoding. Encoding context establishes a deeper level of relationship between an item and its contextual features, providing a distinctive set of cues to separate it from other information, and preventing confusion and competition at retrieval (Badre & Wagner, 2005).

Research has consistently demonstrated greater PI with increased similarity of learned information (Bunting, 2006; Underwood, 1957; Wickens, Born, & Allen, 1963). Because the MOL encourages distinctive encoding, the similarity between to-be-learned items decreases. Taken together, the MOL should reduce PI relative to strategies without distinctive cues. We predict that the MOL will reduce proactive interference due to decreased similarity between learned items.

Keppel and Underwood (1962) suggested that the amount of PI is directly related to the number of potential interfering associations such that more previously learned associations result in more PI. In other words, it is more difficult to select one option from among its competitors when incompatible responses are associated with the same cue. When this happens the result is cue-overload or simultaneous competition for response between multiple candidates, which produces interference (Ikier et al., 2008; Jonides et al., 2008; Postman & Underwood, 1973). Evidence for the principle of cue-overload is found in studies of sentence comprehension in which the availability of any given item decreases as the probability that its cues match another item increase (Van Dyke & McElree, 2006). Also, De Beni and Cornoldi (1988) found that the MOL was less effective for congenitally blind participants as the number of items associated with a particular location increased. Because blind participants have no visual image, a single locus does not have various cue sources on which to-be-learned information can be integrated. This finding is consistent with a cue-overload approach in interference literature.

The PI effect has been described as an overload on retrieval cues (Wickens et al., 1981), and becomes apparent when irrelevant, overlapping, or similar information is encoded with target information and is activated at recall with the target information creating competing intrusions and lapses in memory (Hasher, Tonev, Lustig, & Zacks, 2001). If the encoding of contextual information is compromised, then so is the selection of appropriate context alternatives at recall. By inhibiting the initial access to marginal information, which may be a benefit of the MOL, one can improve encoding specificity, increasing inhibition of interfering material at retrieval and reducing competition (Hasher, Stoltzfus, Zacks, & Rypma, 1991).

PI has traditionally been thought to occur at retrieval (Postman & Underwood, 1973; Wickens et al., 1981). The suppression of competing information at recall and its resulting release from PI was noted by Wickens et al. (1981) and has become the cornerstone of the inhibition-reduction theory. This theory suggests that PI results from inadequate inhibition of task-irrelevant information (Hasher & Zacks, 1988; Lustig, Hasher, & Zacks, 2007). From this perspective, the information most likely to cause PI arises from secondary tasks that involve the same domain of information as the primary task, as was demonstrated in working memory span tasks (Lustig, May, & Hasher, 2001). Inhibition or suppression focuses attention on task-relevant information by constraining initial access to marginal information and by restraining strong but irrelevant responses triggered by familiarity (Hasher et al., 2001).

The purpose of our study was to determine if the proactive use of the MOL will reduce PI on successive word lists drawn from the same semantic category relative to spontaneous encoding/retrieval strategies. Because the MOL enhances distinctive encoding, similarity decreases and retrieval competition between target and non-target information is likely reduced. We predicted PI for both the no-instruction and MOL groups, but lower PI for participants trained on the MOL. We also predicted that the MOL would result in higher overall recall compared to no instruction.

## Method

### Participants

Ninety-four students (*M*_age_ = 19 years) from an undergraduate introductory psychology course at California State University, Fresno, participated in this experiment, each receiving one credit toward course requirements. We collected data from 94 students because that many participants signed up for the experiment through an on-line experiment system.

### Materials

A list of similar words from the category of fruits found in a category of norms by Battig and Montague (1969) was selected, sorted into blocks of five words of descending frequency, and randomized into five sets of five words. Each list was counterbalanced by word frequency so that it had a balance of the most and the least frequently occurring words. The lists were then balanced by word length so no list had excessively short or long words. Finally, the lists were balanced so each list had only one citrus fruit (see Appendix A for stimulus lists). The first and fifth lists were counterbalanced by list position (that is, List 1 was moved to the List 5 position, and List 5 moved to List 1 position) so any effect observed would not be due to one individual list being easier to recall than another.

### Procedure

A free recall paradigm involving word lists of a single category was used to investigate the effectiveness of the MOL on similar-item list recall. We expected that lists of semantically similar items would increase PI across these lists (Wickens, 1970). The strategy instruction (none vs. MOL) was manipulated between-subjects. The serial position of the list (first list vs. last list) of the five remembered lists was treated as a within-subjects variable. Proportion recall was the single dependent variable.

Participants were tested in groups of three to 15, with each group randomly assigned to a no-strategy-instruction (NSI) condition (*n* = 48), or MOL condition (*n* = 46). Mean demographic characteristics and vocabulary scores (collected at the end of the experiment) are presented in Table 1, showing no a-priori differences across instruction groups.

**Table 1.** Mean Demographic Characteristics and Vocabulary Assessment in the No-Strategy-Instruction (NSI) and Method-of-Loci (MOL) Groups

Group	Age	Education	Gender %		Voc.	Wellness
	Years	Years	Male	Female	%	%
NSI	18,8 (1,1)	12,8 (0,9)	35	65	62 (9,4)	70 (17,0)
MOL	19,1 (1,3)	13,4 (1,3)	37	63	65 (9,9)	76 (18,7)

*Note*. Voc. = vocabulary. Standard deviations are in parentheses.

Without any reference to strategy, the NSI participants were instructed to remember the presented words. The MOL participants received instruction on the MOL immediately prior to stimulus presentation. The experimenter first gave an overview of the MOL then showed a 5-min video by British memory champion, Andi Bell (BBC, 2007), on the use of imagery in memory and the MOL. Because recall is stronger for subject-generated loci in an expository passage (Moe & De Beni, 2005) and for subject-generated images (Mulligan, 2004), participants in the MOL group generated their own loci and images using their own homes. They were instructed to pick five places in each of five adjoining rooms making a pathway or a series of 25 loci. These loci were already well known and required no memorization. To insure that all participants had a clear mental pathway, they were asked to diagram the pathway through their house, naming each place. Each room would hold one list, and each locus within the room would hold one word. The rooms functioned to separate words lists, and the loci within the room separated words on the list. Participants were briefly instructed to create an image of the interaction of each word with its locus when the items were presented. The instructions were simple and relied on the participants’ initiative and ability to implement the strategy. Immediately following this 25-min instruction period in the MOL, the room diagrams were turned over and were not used for reference during either learning or testing.

All participants viewed and heard (via associated pre-recorded audio) five lists of five words (or a total of 25 words) from the fruit category using PowerPoint software. The words were presented both orally (from pre-recorded audio embedded in the slides) and visually at a rate of one word per 10 s. Other list studies have used from 2- to 14-s encoding periods, however the amount of time allowed does not appear to be as important as how it is used (Lustig & Flegal, 2008). Each list was followed by a 40-s basic math distractor task to prevent rehearsal before recall. This task included mental addition, subtraction, and multiplication with answers only being recorded in a student data booklet.

Following the distractor math task, participants had a 20-s period in which they were asked either to write the last list they had seen (for Lists 1 and 5) or to complete a second set of math problems (for Lists 2-4). Based on pilot testing, this time was sufficient to recall and write the five words. All participants were given recall instructions only after the first and last lists. They were told to expect testing on some but not all lists and that they would not know which lists they would be tested on. Testing on the first list provided a baseline of recall before PI built up and testing on the fifth list provided a measure of recall in a condition of high PI in which the effectiveness of the MOL could be tested. PI has been shown to build up as word lists of similar items activate multiple retrieval choices (Wickens et al., 1981). No testing was done on the intervening lists because PI is resolved by frequent testing (Szpunar, McDermott, & Roediger, 2008), and our goal was to build up maximal PI before testing participants on recall of the final five words.

Participants were told it was important that they invest effort to learn each list because they would be asked to recall all of the words on a final test at the conclusion of the study. Immediately after recallingthe items from List 5, participants were instructed to turn their recall packets to the next (blank) page and write down all the words from the experiment in any order. They were given 2 min to complete the final testing. All participants then completed a questionnaire with information on age, education, learning mode, gender, well-being (on a 10-point scale), and their own use of strategy (using an open-ended question). Finally, they completed a 42-item Shipley Vocabulary Index and were fully debriefed.

## Results

### Initial testing

**Figure 1.**
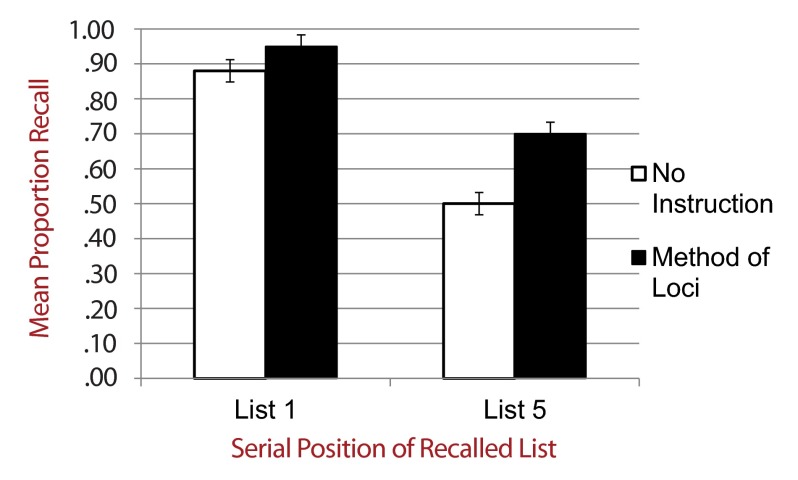
Mean proportion correct free recall by serial position of recalled list and encoding-retrieval strategy instructions.

All statistics were two-tailed with an alpha level of .05. A two-way analysis of variance (ANOVA) was conducted on proportion recall of five words across Strategy Instruction (none vs. MOL) and Serial List Position (first vs. last). Table 2 includes all means and standard deviations in each condition. Figure 1 shows mean proportion recall across all conditions. A main effect of instruction was found such that proportion recall in the MOL group (*M* =.82, *SD* = .21) was higher than in the NSI group, *M* =.69, *SD* = .23, *F*(1, 92) = 13.89, p < .001, η_p_^2^ = .13.Also, a main effect of list position was found such that proportion recall in List 1 (*M* = .91, *SD* = .16) was higher than in List 5, *M* =.60, *SD* = .31, *F*(1, 92) = 104.46, *p* < .001, η_p_^2^ = .53. There was an interaction between Strategy Instruction and List Position, *F*(1, 92) = 4.82, *p* < .05, η_p_^2^ = .05,showing a greater proportion recall difference between strategy instruction groups in List 5 than in List 1.

**Table 2.** Mean Proportion of Words Recalled for the No Strategy Instruction (NSI) and Method of Loci (MOL) Groups on List 1 and List 5

Group	Words recalled		PI index
	List 1	List 5	
NSI (*n* = 48)	.88 (.18)	.50 (.27)	.38*
MOL (*n* = 46)	.95 (.11)	.70 (.31)	.25*

*Note*. PI = proactive interference. Mean proportion of words recalled out of five words in each list. Standard deviations are given in parentheses. *One-sample *t*-test compared to 0, *p* < .01.

Data were also analyzed by a PI index, calculated for each participant by subtracting List 1 proportion recall from List 5, with higher positive numbers indicating greater PI. Two one-sample *t*-tests were run against the test value of zero to determine PI build-up in each group. The results indicated the presence of significant PI in both the NSI group, *M* =.38, *SD* = .30, *t*(47) = 8.86, *p* < .001; and the MOL group, *M* =.25, *SD* = .30, *t*(45) = 5.63, *p* < .001. In other words, control participants who had NSI exhibited a 38% decrease in the number of words across lists, and participants instructed in the MOL exhibited a 25% decrease across lists. An independent groups *t*-test demonstrated that the mean PI index for the NSI group was significantly higher than for the MOL group, *t*(92) = 2.19, *p* < .05, *d* = 0.45, demonstrating that PI was greater in the NSI group than the MOL group.

Planned comparisons using independent groups *t*-tests showed hi-gher proportion recall for List 1 in the MOL instruction group (*M* =.95,*SD* = .11) than in the NSI group, *M* =.88, *SD* = .18, *t*(92) = 2.17, *p* < .05, *d* = 0.45; and for List 5 between MOL (*M* =.70, *SD* = .31) and NSI groups, *M* =.50, *SD* = .27, *t*(92) = 3.41, *p* < .001, *d* = 0.70. Together, these results indicate that the MOL instructions enhanced recall across serial list positions relative to no instruction.

### Final free recall test

**Figure 2.**
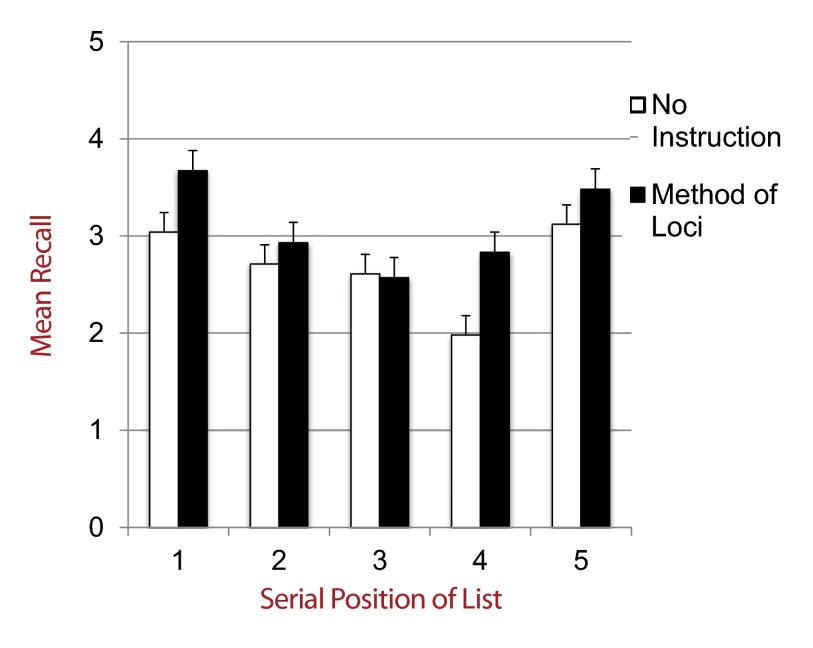
Mean correct recall on final free recall test out of five items by list and encoding-retrieval strategy instructions.

For the final comprehensive test of all list items, an independent groups *t*-test revealed significantly higher recall for those instructed with the MOL across all lists (*M* =15.52, *SD* = 4.54) than NSI, *M* =13.60, *SD* = 3.34, *t*(92) = 2.34, *p* < .05, *d* = 0.48. Figure 2 shows final recall across all conditions.

#### Previously tested items

To investigate whether the effects of PI persisted across time, we compared initial recall of Lists 1 and 5 to recall of those list items on the final test. Two one-sample *t*-tests showed no significant PI (as measured by the PI index) in either the MOL, *M* =.04, *SD* = .38, *t*(45) < 1; or NSI, *M* =-.03, *SD* = .39, *t*(47) < 1, conditions. This indicates no measurable PI in the final free recall test. A closer analysis using paired-samples *t*-tests revealed that this was due to reduced recall on List 1 items in the MOL condition from initial testing (*M* =4.74, *SD* = 0.57) to the final test, *M* =3.67, *SD* = 1.43, *t*(45) = 5.03, *p* < .001, *d* = 0.98. There was no significant difference on MOL recall of List 5items from initial (*M* = 3.58, *SD* = 1.45) to final testing, *M* =3.48, *SD* = 1.49, *t*(44) < 1. For the NSI condition, the lack of PI was due to both reduced recall on List 1 items from initial testing (*M* =4.40, *SD* = 0.92) to the final test, *M* =3.04, *SD* = 1.56, *t*(47) = 5.50, *p* < .001, *d* = 1.06; and to increased recall on List 5 items from initial testing (*M* =2.48, *SD* = 1.37) to the final test, *M* =3.17, *SD* = 1.15, *t*(47) = 3.79, *p* < .001, *d* = 0.55. This suggests a hypermnesic effect (see Payne, 1987), perhaps due to a release from PI on the final test in the NSI condition.

#### Previously untested items

To assess whether MOL training affected final recall across the untested lists, we conducted a two-way mixed-subjects ANOVA by Strategy Instruction (MOL vs. NSI) and List Position (2, 3, 4). We analyzed recall only from Lists 2-4 because participants had already attempted to recall Lists 1 and 5 during initial testing. Here, there was no main effect of instruction, demonstrating no difference in final recall of Lists 2-4 between MOL strategy instruction (*M* =2.78, *SD* = 0.98) and NSI, *M* = 2.45, *SD* = 0.98, *F*(1, 92) = 2.54, *p* > .10, η_p_^2^ = .027. However, there was a significant main effect of recall across List Positions 2, 3, and 4, *F*(2, 184) = 3.26, *p* < .05, η_p_^2^ = .034; and a significant interaction between Strategy Instruction and List Position, *F*(2, 184) = 4.31, *p* < .05, η_p_^2^ = .045. As demonstrated in Figure 2, this interaction was driven by List 4, showing higher recall in the MOL condition (*M* =2.83, *SD* = 1.32) than NSI, *M* =1.98, *SD* = 1.33, *t*(92) = 3.10, *p* < .01, *d* = 0.64. Although there was no main effect of strategy instruction, these analyses suggest that PI built up across lists and was measurable by List 4 on a final recall test.

To assess whether the difference in List 4 recall was merely due to output interference in the final free recall test, we calculated an output interference index by first counting the number of words recalled before each List 4 item, then calculating a mean for each participant. Participants who did not recall a List 4 item were not included in this analysis. Because more List 4 items were recalled in the MOL condition, we divided the number of previously recalled items’ mean by the total number of List 4 items recalled for each participant, thereby calculating a scaled output interference List 4 index for all participants. (Three MOL participants and five NSI participants recalled no items from List 4 and were not included in the analyses.) An independent groups *t*-test demonstrated a significantly higher output interference index in the MOL condition (*M* =9.42, *SD* = 4.37) than NSI, *M* =6.31, *SD* = 3.20, *t*(84) = 3.76, *p* < .01, *d* = 0.81. This demonstrates that output interference was higher with MOL than NSI, thereby eliminating the explanation that lower List 4 recall with no strategy in the final free recall task was due to output interference.

#### Output order

To assess whether participants used a forward serial recall strategy on the final recall test, we calculated an Asch-Ebenholtz (AE) index of forward seriation (Asch & Ebenholtz, 1962) for each participant. This measure assesses the match between input and output order with values ranging from 0 to 1, with 1 indicating a forward serial match and .5 indicating random recall with no match between the presentation and recall order. For example, across the 25 words presented, if a participant recalled six words all in the same relative forward serial order (e.g., Words 2, 8, 9, 11, 17, 23, in that order) the AE index for that participant would be 1.0. In essence, an AE index measures the degree to which a participant’s recall follows the same relative serial position as the presentation order. We calculated this index to assess whether participants trained on the MOL were more likely to use forward seriation on the final free recall test. An independent groups *t*-test demonstrated higher mean AE with training on the MOL (*M* =.70, *SD* = .20) than NSI, *M* =.53, *SD* = .12, *t*(92) = 4.97, *p* < .001, *d* = 1.02, thereby indicating that MOL participants had a higher input-output forward seriation match. Although we do not know what strategy participants used at recall, these data suggest that participants trained in the MOL were more likely to recall items in forward serial order - the same order that forms the basis of the sequential strategy in the method of loci.

### Personal strategies

To investigate what spontaneous, personal strategies were used by participants in the NSI control group, we descriptively analyzed self-report of the described strategies. All participants reported using some type of personal strategy to recall word lists (see Table 3). Rehearsal and first letter strategies (average PI Index of .45) were the most commonly reported strategies used and accounted for 63% of the participants in the NSI condition. Story or image, which are most similar to the MOL were used by only 13% (6 of 48) of the participants, but resulted in a small difference in mean recall between Lists 1 and 5. We are hesitant to infer anything from these data given the small sample.

**Table 3.** Comparison of Spontaneous Strategies Reported in the Control Condition Without the Method of Loci

Strategy	Image (*n* = 6)	Rehearse (*n* = 21)	First letter (*n* = 9)	Experience (*n* = 7)	Unclear (*n* = 5)
L1 word recall	4,50	4,14	4,89	4,43	4,40
L5 word recall	4,16	1,86	2,78	2,29	3,00
Difference L1-L5	0,33	2,29	2,11	2,14	1,40
PI Index	.06	.46	.42	.43	.28

*Note*. L = list. PI = proactive interference. N = 48.

### Intrusions

In the current context, intrusions can provide useful information about information that might be inhibited as a result of retrieval competition. However, across all participants in all conditions, the mean intrusions were very low (*M* =0.35, *SD* = 0.82), indicating floor effects on intrusions. An independent groups *t*-test across strategy condition, collapsed across list, indicated no difference between mean intrusions in the MOL (*M* = 0.39, *SD* = 1.04) and NSI conditions, *M* =0.31, *SD* = 0.62, *t*(92) < 1. Overall, these intrusion data are not surprising given that recall was only out of five items for each list.

### Demographics

To exclude the possibility that any findings might be attributed to a demographic confounding variable, a series of independent samples *t*-tests revealed no significant differences in vocabulary, age, or well-being. However, this does not preclude the possibility that a confounding variable still existed. With our randomization procedures, stimulus counterbalancing, and demographic information, the likelihood of a confounding variable is greatly reduced.

## Discussion

In an era of high distraction, increased lifespan longevity, and increasing concerns about memory failures, strategies that might reduce forgetting are increasingly important. The current study’s aim was to investigate whether PI - a main cause of forgetting - can be reduced by using the mnemonic strategy of the MOL, well known to enhance retention through the proactive use of distinctive cues. PI is reduced through proactive strategies in directed forgetting (Bjork & Bjork, 1996; Gazzaley et al., 2005; Sahakyan & Delaney, 2005) in which PI is reduced by the instruction to forget a non-target list and also in observations that expecting PI results in less actual PI (Braver, Gray, & Burgess, 2007; Ikier et al., 2008). The point here is that previous research demonstrates reduced PI by intention to forget or expectations. Here, we found that PI can also be reduced by implementing an intentional mnemonic strategy.

It was necessary to first establish that our procedure caused measurable PI. With exposure to multiple exemplars in the same semantic category across five lists, we found a 38% reduction in recall from List 1 to List 5 in our control group who encoded and retrieved the words using their own idiosyncratic strategies. Additionally, and perhaps more importantly, we found that MOL training resulted in a significant reduction of PI relative to NSIs, with only a 25% reduction in recall across lists. The issue addressed in this study is most accurately described as a comparison of the proactive use of the MOL with other spontaneous, personal strategies in the control condition; and it demonstrated that the MOL reduces PI relative to spontaneous strategies.

It is also noteworthy that the benefits from MOL training extended to a free recall task in which participants were only given the instruction to recall the items in any order. This test reflected a recall delay of about 10 min from the presentation of the first list, showing the benefits of earlier training without any instruction to use the MOL. Evidence that participants trained on the MOL were still relying on it to a certain degree during the final test is shown by greater forward seriation compared to NSI, as measured by the AE index.

As mentioned, retrieval competition between to-be-recalled items is a common explanation for PI. Output interference is a type of PI that refers to the deleterious effects of retrieval on the subsequent retrieval of other information (e.g., Smith, 1971; Tulving & Arbuckle, 1963) and has been used as an explanation of why free recall ceases, even when information is still available in memory (Rundus, 1973). In other words, retrieval causes temporary inaccessibility to previously-learned information. Framing this as a cue-to-item search, as items are retrieved, those items are strengthened relative to the other items. When searching for additional items, the recently-retrieved item is more accessible, thereby interfering with retrieval of those additional items (see Raaijmakers & Shiffrin, 1981). Consistent with this, Smith (1971) found greater output interference as semantic category size increased. In the current study, each list is associated with a single location using the MOL. In essence, the MOL creates an additional hierarchy level, thereby reducing the number of target items associated with any single cue relative to using a single category cue (in this case, the category of “fruit”). This provides an explanation for the mechanism of reducing response competition and subsequent PI by using the MOL.

One way to assess the buildup of PI and whether non-target information is suppressed is by analyzing extralist intrusions (e.g., Hasher, Chung, May, & Foong, 2002). However, our intrusion rates were very low (i.e., mean of 0.35 intrusion per five-item list). This could be a function of our procedure (i.e., recalling 10 exemplars of one semantic category) or it may indicate a lack of response competition. Intrusions are a secondary measure of PI and previous research often does not include a discussion or analysis of intrusions (e.g., Badre & Wagner, 2005; Wickens & Clark, 1968; Wickens et al., 1976). Future research might employ procedures that increase intrusions, allowing a direct analysis of response competition predictions.

Using the MOL, participants imagined each word list in a separate room as they heard it. In studies involving word list recall, there is evidence that each list is both encoded in long-term memory and retrieved from long-term memory as a set of words, suggesting that interference effects involve a response set (Wickens et al., 1981). Visualizing each list in a different room may help to separate the list as a set. Participants were instructed to visualize each word at its own location within that room. In this way contextual and visual cues for each word would be distinctively bound to a unique place. Importantly, we note that PI was reduced using different loci for each list and unique holders for each item within that location. We do not know whether PI would be reduced without those unique visual pegs.

Hedden and Yoon (2006) found that visual memory can significantly contribute to the ability to resist proactive interference. Imagery provides an immediate way to integrate a set of information (Rubin, 1995) and it is well suited for moving quickly from one situation to another (Paivio, 1971, as cited in Rubin, 1995), but it is the loci that hold items in memory and make items more accessible in recall (Massen & Vaterrodt-Plünnecke, 2006; Roediger, 1980). Image appears to function locally to improve recall by accessing only local information at one time in one place (Rubin, 1995). Together with loci, image constrains the number of competing possibilities of memory representations by inhibiting the choice of extraneous information. However, this does not preclude the possibility that any imagery-based mnemonic might show the same pattern.

Our results further show that when the MOL is used to learn word lists, forgetting is reduced (or retention is enhanced) by 7% on the first list and by 22% on the fifth list. Wickens and Clark (1968) found a release from PI by changing the conceptual class or semantic category of words. In the final free recall task, we found no differences between List 1 and 5 recall in either condition, suggesting that PI was released in both conditions. This may have been due to previous testing of those materials (see Szpunar et al., 2008). For previously untested lists, the final free recall test demonstrated a significant decline in recall across serial list position for NSI but not for MOL participants, thereby suggesting lasting PI for NSI participants.

An interesting observation based on self-report of our participants is that most of them used some type of strategy to learn and recall the word lists. Most (94%) of those who were instructed in the MOL reported making the effort to use it with varying degrees of confidence. Spontaneous strategies were reported by 43 of the 48 NSI participants. Descriptions of spontaneous strategies in the participant self-report fell into four general categories. Imaging included visualizing the fruits or creating a story and was used by 13% (*n* = 6) of NSI participants. This category showed a PI index of .06 - the lowest of the four categories and the most similar to the MOL. We hesitate to make strong inferences on these data given the small sample.

The most common reported strategy was rehearsal, typically described as repeating words over and over, or repeating the fruits by color, and was used by 44% (*n* = 21) of NSI participants. The PI index for this category was .46. Nine participants (19%) used a form of first-letter strategy by making an acronym of first letters or using the first syllable, resulting in a PI index of .42. Finally, seven participants (15%) reported reliance on personal experience to remember words (e.g., thinking of favorite, interesting, or good tasting fruits), resulting in a PI index of .43. Five participants (10%) did not provide a spontaneous strategy description and had a PI index of .28. This distribution of free recall strategies parallels patterns found in previous research (Delaney & Knowles, 2005; Sahakyan & Delaney, 2003). In comparing the MOL use to these various strategies, we found that the PI index for rehearsal was nearly twice that for the MOL and it was the most common spontaneous strategy, used by 44% of the NSI group. Our participant self-report suggests that while spontaneous strategies are frequently used to aid recall in situations that are likely to cause PI, they are less likely to be as effective as the proactive use of a strategy like the MOL (or perhaps any strategy that involves imagery). However, we are cautious about making strong inferences here, as these strategies were evaluated and categorized from open-ended responses.

Some studies have shown a preference for certain types of strategies. Individuals with high attention control tend to favor elaborative strategies during encoding while low attention individuals are more likely to use rehearsal strategies; the difference in encoding strategies may account for the greater resistance to PI among those with higher attention control (Cokely, Kelley, & Gilchrist, 2006). In a study of those with superior memories, all used memory strategies, and 90% reported using the MOL (Maguire, Valentine, Wilding, & Kapur, 2003). Memory training studies affirm the strength of this strategy to improve memory performance in both young and older participants (Lustig & Flegal, 2008; Massen & Vaterrodt-Plünnecke, 2006; Nyberg et al., 2003; Rebok & Balcerak, 1989). In our study, participants without instruction in the MOL recalled about 88% of the low interference List 1 and about 50% of the high interference List 5. However, those with the MOL recalled about 95% of the words from the low interference List 1 and about 70% of the high interference List 5.

Although the data suggest that MOL training reduced PI, we cannot rule out the possibility that simply having a 25-min training session, regardless of its content, had the unintended consequence of enhancing recall and/or implicitly decreasing PI. That is, these data might be explained by practice, enhanced attention, expectations, or anxiety reduction due to the training session itself. However, an explanation based on a confounding variable seems inconsistent with the recall increase on List 5 from initial to final testing in the NSI condition. Additionally, there was no difference in recall across MOL and NSI for previously-untested lists in the final recall task. Future research should directly compare MOL training with other mnemonic strategies or an irrelevant training task to eliminate possible confounds and to assess the effect of MOL relative to other mnemonic strategies. We also acknowledge that the effect of PI across strategy conditions may be confounded with other factors that affect memory. If this were true, given the history of PI research using similar procedures, it would likely be driven only by providing a mnemonic strategy. Future research might investigate this by including a control condition of categorically-unrelated items.

*Hypermnesia*, a concept rooted in the literature of spontaneous recovery of extinguished animal behaviors, refers to increased recall across successive recall attempts (Payne, 1987). This is consistent with the finding that information can be available (i.e., sufficiently encoded) but inaccessible (Tulving & Pearlstone, 1966). Comparing recall of List 5 items in initial testing (in which there was maximal PI) to recall in the final free recall test of all items, we found no recall difference in participants with MOL training. However, we found a significant recall increase for participants who received NSI from initial to final testing, thereby demonstrating hypermnesia. Research has suggested that hypermnesia may partially be caused by a release from interference (Madigan & Lawrence, 1980; Tulving & Psotka, 1971) - an interpretation consistent with our conclusion that greater PI occurred in the NSI condition.

Interference theory provides a general framework for analyzing the processes of forgetting (Postman & Underwood, 1973). According to this theory people fail to remember not because information is lost, but because other information obstructs access to target information. Organizational mnemonics such as the MOL evolved to meet this very need in a culture that had to depend on memory in daily societal life and as a means to pass on their history. The MOL organizes information and sets it in a memory structure where it can be accessed like items in alphabetically arranged files in a drawer. However, mnemonic strategies are proactive and must be intentionally used with forethought and preparation. The mental structure, as simple as it is, must first be learned, and a conscious decision must be made to use it in a given situation where forgetting is likely. Effort and visual ability are involved in associating images at each location, as was noted by participants in this study. But when that is done, even with only 25 min of instruction and loci map making, the MOL can equip a person to diminish the specific memory hazard of proactive interference. This is particularly important given that the MOL can be repeatedly used effectively (Massen & Vaterrodt-Plünnecke, 2006). Future research should explore this mnemonic as a way of reducing PI in populations that are particularly susceptible to the buildup of proactive interference, such as those with traumatic brain injury or older adults.

## Appendix A

Stimulus lists

List 1/5: *cantaloupe, lemon, mango, pineapple, pear*

List 2: *apricot, raisin, plum, orange, raspberry*

List 3: *strawberry, tangerine, fig, banana, cherry*

List 4: *pomegranate, avocado, grapefruit, peach, prune*

List 5/1: *blueberry, watermelon, apple, grape, lime*
